# Genealogy, critique, and decolonization: Ibn Khaldun and moving beyond filling the gaps

**DOI:** 10.12688/openreseurope.16148.1

**Published:** 2024-01-10

**Authors:** Sertaç Sehlikoglu

**Affiliations:** 1 Institute for Global Prosperity, University College London, London, England, W1T 7NF, UK

**Keywords:** Decolonising, Anthropology Theory, Genealogy, Critique

## Abstract

The aim of this paper is to locate critique at the intersections of the genealogy of knowledge in anthropological thinking and the decolonizing movement. The paper approaches the decolonizing movement as one of the most crucial points in anthropological thinking. It is built on the premise that the decolonizing movement is set to go beyond filling the gaps in genealogies and it can do so by: (1) revising the ‘dismissed’ genealogies that has contributed to the formation of the contemporary classical theory, and (2) thinking creatively in implementing the critical thinking tools to the dismissed scholarship, in an equal manner to the Eurocentric scholarship. To illustrate, it uses the case of Ibn Khaldun, an Arab scholar of social sciences and historical analysis from 14 ^th^ Century who is often referred to as the first sociologist. On the one hand, his influence in classical Western thinking is largely dismissed. On the other hand, as a counter-response to this dismissal, the new Islamic revivalist intelligentsia in the Muslim right engage with him in a selective manner that not only rejects that central critical thinking but, even worse, sanctions the local regimes of power, including that local canon. By locating his scholarship to multiple tropes in anthropological theory and reading his evolutionist thinking vis-à-vis the post-colonial literature in anthropology and sociology, I question the limits and possibilities of critical thinking within and beyond the decolonizing movement.

## Introduction

The aim of this paper is to locate critique at the intersections of genealogy of knowledge in anthropological thinking and the decolonizing movement. I connect a two-tiered set of questions as a way to inquire about the limits and possibilities of critical thinking within and beyond the decolonizing movement.

In the first tier, the paper criticizes the series of work in anthropological theory that tends to somehow start their genealogies with the Ancient Greek thinkers and then jump for about two millennia and continue with enlightenment, omitting all the debates, conversations, and disagreements (the three main components of the evolution of social thought) that took place in between. This leap, in other words, involves a dismissal to the series of scholarly conversations and advancements that took place from 400 BC to the 1500s across the Mediterranean Sea. Then, in this first layer, I engage with the interconnected questions of intellectual impoverishment due to omission and questions on method of engagement. I, therefore, bring in one of the many dismissed scholarships with crucial value to the genealogy of knowledge we today claim as Western: the Classical Arab Scholarship. By Classical Arab Scholarship, I refer to the substantial number of works written in Arabic and circulated across North Africa, the Middle East, and parts of East Asia from roughly the late 900s to early 1400s. The word Arab in this category of Classical Arab Scholarship does not refer to the ethnic background of the scholars, neither does it necessarily signify any particular religion. Rather, it is used to refer to the language in which the individuals from various ethnic backgrounds were trained and had authored their work.

In this first layer, I ask: What happens when a massive chunk of rich scholarly developments and conversations, which took place for as long as 700 or so years, is removed from our sense of literature, our readings of the resources, including both the classical and ancient texts. What it means to have interruptions in this genealogy? What epistemological scope does this selective reading and referencing bring to our understanding of knowledge? Should we address such substantial gaps and, if so, how should we do that? I am not answering each one of those questions in this paper but laying them out to warm up. What I do in this first tier is approach classical Arab scholarship as both Western and non-Western at the same time.

In the second tier, I then question the “how” of the decolonizing curriculum. My main question there would be whether and how we are to engage with the native scholars in anthropology and with their theories. Bear in mind that some of those theories were part of the genealogical knowledge productions but were today dismissed. So my inquiry then inevitably has another lingering effect: is filling the gaps enough to decolonize?

To expand the layers of this second point, I take a closer look at a particular figure in classical Arab scholarship, Ibn Khaldun and his theory of the state and state-making in relation to power. I critically engage with the ways in which he is appropriated by various local regimes of power in the Middle East and various Western romantic views.

The topic “decolonizing movement” is highly contested and thus, it is important clarify from the beginning that this paper does not claim a universal truth or principle about how to decolonize. What it does very clearly however is take a denunciatory position against the contexts where the decolonizing movement is appropriated in non-Western contexts to silence the critical voices. Therefore, the paper also touches on the cases where the new Islamic revivalist intelligentsia in the Muslim right engage with, praised, and even used Ibn Khaldun in a selective manner that paradoxically both: a) establishes a canon voice that marginalizes critical voices, and b) directly or indirectly serves to the local regimes of power.

In the conclusion, I complete my circular thinking by returning to the question of “dismissal” as a failure of scholarly diligence and intellectual imagination.

## Selected debates on decolonising anthropology

It would be helpful to start with reflecting on what it means to decolonise knowledge in general and decolonize anthropology in particular.

With the help of Foucauldian theory on power and knowledge, anthropological knowledge has been criticized, especially in the 80s and 90s, as a discipline which emerged for Western colonization and as a result of Western colonization. The significance of the postmodern turn in anthropology was that it has stimulated a wave of thinking that questions the legitimacy of various sciences and social sciences as knowledge making mechanisms. 

For the sake of staying in the nuanced side of the conversation and of the probe, it is important to remind ourselves one more concern in the formation of anthropology as a discipline. Anthropology as a discipline was initially established to form scientific knowledge about the colonially encountered other (and of course through other about themselves). Since a large number of early anthropologists were financially supported by the colonial officers (if not acting as one), during the periods preceded the establishment of anthropology as a discipline, the knowledge produced about this encountered “other” was through the travel notes of the diplomats.

A considerable number of those popular works were about places they have never been to and the people they have never met. Just to give one single example, Richard Knolles published his book “General History of the Turks” in 1603, without a single visit to the geography he wrote about. The book became so popular that it had seven editions.

Establishing a discipline that will use scientific standards of knowledge production to study the newly encountered societies was therefore one of the rationales behind the need for a discipline like anthropology. And here is the crucial point: What is considered as ‘scientific’ had not been proven to provide a reliable ground for this new discipline. The scientific requirements to be met at the time were highly problematic. Namely, cultural evolutionism and the preoccupation for cataloguing various races to fit into this unilinear sense of social progress embedded in cultural evolutionism, had later become abandoned practices and methods specifically for promoting what we today call scientific racism. Written when cultural evolutionism was considered to be an accepted fact, the language used in early anthropological texts assumed racial, social, and intellectual superiority of the industrialized societies and thus, referred to Aboriginal and indigenous groups as *primitives*, and even *savages*. 

From the perspective of the decolonizing movement, therefore, the issue at stake was more than having racist undertones that “could” be discussed whether they were actually racist or not. The issue at stake is how several of those works have actively contributed to the production of a knowledge that suggests and even ranks racial hierarchies.

The postcolonial theory has challenged the scholarly legitimacy of these early work. The decolonising movement takes a more radical position, however. The decolonising movement has emerged out of a disappointment and frustration with the way the curriculums are formed, and citation politics are shaped in a manner that still does not address the native scholars’ critical interventions. The dismissal of critical interventions meant attempting to continue canonical writing as if, for instance, Frantz Omar Fanon never reminded us of the centrality of race and eurocentrism in psychoanalysis and studies on self, Edward Said never wrote hundreds of pages to demonstrate English literature is not solely English, Gayatri Spivak has not introduced the terms essentialism and strategic essentialism to our vocabularies. The very dismissal therefore has simultaneously resulted postcolonial theory to be treated as ‘fads’ to be moved on and moved away from. As a response to this wider problem of citation politics and canonical writing, the decolonizing movement has emerged believing that a more fundamental position is required, by replacing the existing dismissive canon with the scholarship of the marginalized native scholars.

While those who support and join the decolonizing movement were taking issues with the anthropological canon for being dismissive to the native scholars, the older generation of scholars who form the anthropological canon had another concern. Their concern was about how to keep the anthropological canon in the canon. But then, how would we separate what is anthropological apart from what is not?

On 31 ^st^ of August in 2017, the renowned anthropologist Marshall Sahlins published a blurb on the HAU Facebook page where he asked “Where Have All the Cultures Gone?”. He starts by asking: “What happened to anthropology as encompassing human science? Why is a century of the first-hand ethnography of cultural diversity now ignored in the training and work of anthropologists?” He questions the way contemporary anthropology has started dismissing its own foundational knowledge.

Sahlins then continues with listing several examples that he believes to be removed from the recent anthropology curriculum including Naga head-hunting, Fijian cannibalism, Amazonian animism, and Aztec human sacrifice. All of these topics are those I teach and show images with a viewer discretion and a courtesy warning, in line with contemporary pedagogies sensitive to diversity and inclusivity. What Sahlin suggests however is that anthropologists are “the custodians of this knowledge, and we are content to let it be forgotten.” With his social media ranting, Sahlins sparked a heated debate within anthropological circles which was quite exciting to follow for a number of us.

Sahlin’s position was the one shared by many traditional anthropologists, especially in the UK but also in the US and Europe. That, all the critical movements and conceptual turns and waves in anthropology have actually hijacked what anthropology should be about. The tension seems to be about the very issue of what anthropology is and should be about. The historical foundation of anthropology as a discipline has shaped both the definition of and critical interventions to anthropology and anthropological thinking. Yet the issue of critique and genealogy rarely goes well with what this paper keeps referring to as dismissal.

It is important to clarify the tension that, the anthropology Sahlins refers to is a foundational anthropology that current theories were built onto and are thus no longer considered to be advanced enough to use in understanding today’s world. Yet, it is equally important to keep the progressive evolutionist understanding in knowledge-making processes under probe. That, our contemporary knowledge is established through the past ones that were not advanced enough, but still have been part of the production processes of today’s what we perceive to be more advanced theories and frameworks.

This paper accepts the main tension in this debate on *genealogy* and the gaps as a productive one and will apply it to the idea of decolonising curriculum in anthropology by using the case of Ibn Khaldun. I join post-colonial theorist Ghassan Hage who, while responding to the conversation in the aftermath of Sahlin’s self-declared rant, that there are two kinds of critique. While some are dominating, silencing, and therefore *paralysing*, some other critiques are continued intellectual discussions, open up new venues and therefore they are *enabling*. In this piece, he called the anthropologists who are involved in the decolonising movement, to do a more productive and enabling critique, of carefully dismantling the problematic elements while staying in conversation with the former generation of scholars. In other words, in Hage’s account decolonising movement is about critiquing the canon but while doing so, creating a more thorough, detailed, stronger and comprehensive genealogies of knowledge.

## Classical Arab scholarship

**Figure 1.**
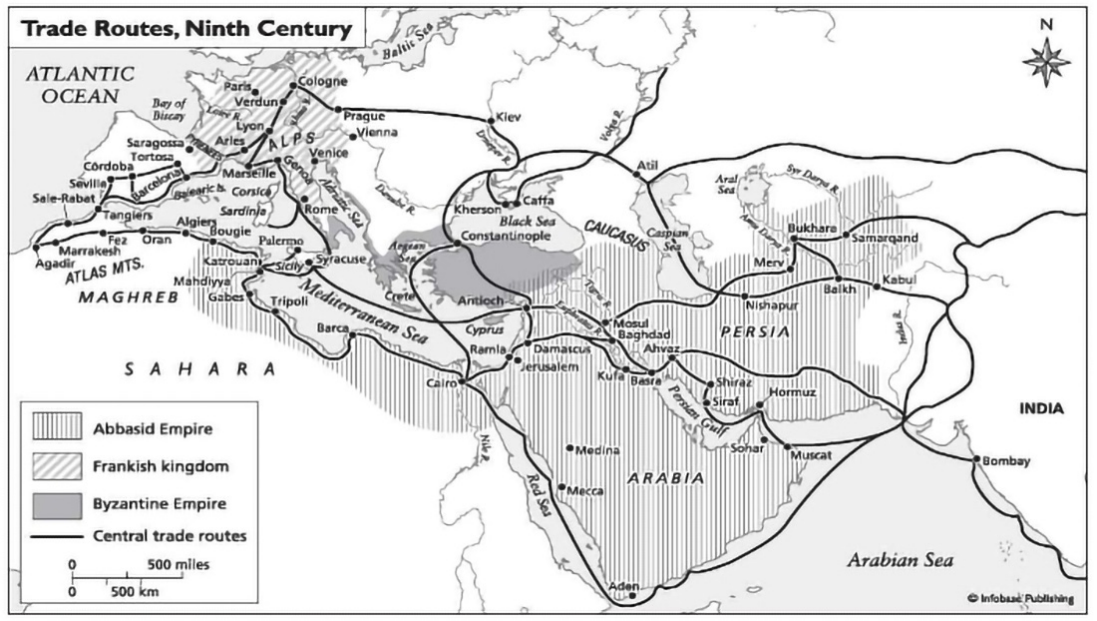
Trade Routes that also shaped the transfer of books and scholarship. Source: http://www.worldhistory.biz/sundries/32597-trade-and-agriculture-under-the-abbasids.html.

If we turn to Classical Arab texts at this point of conversation, we need to remind ourselves that they offer more than simple alternative forms of knowledge. It is important to understand the Classical Arab scholarship as a vast and diverse number of theories and debates that were produced at dozens of institutions connected to each other via trade routes across the Mediterranean and expanding to Central Asia ( Figure 1), which translated and furthered the ancient Greek knowledge of philosophy and sciences. A number of works from this scholarship were later on translated into European languages during the Enlightenment.

I would like to take a moment and repeat my earlier question on why we, anthropologists and social scientists, find it acceptable, if not exciting, to use theories of the ancient Greeks but not the classical Arab scholars. Different approaches suggest different durations, although for at least seven hundred years the ancient Greeks’ work in philosophy, biology, and mathematics were translated, studied and, most importantly, further developed by the Arab scholars ^1^. Surely, in duration of 700 years, there must be some scholarly work worthy of incorporating into our thinking, of engaging with, of giving reference to (even just to disagree)? Why do we skip those works, altogether? What analytical limits we establish through such gaps in genealogy of knowledge?

In fact, today we witness an exciting moment. That we are witnessing a moment with an incredible rise in applying these Classical Arab scholars such as Ibn Khaldun, al-Ghazali, Ibn Arabi, and Albiruni, to core theoretical analysis in the English and French-speaking academia.

A similar rise in interest took place in the 1930s and 1940s mostly in French-speaking academia, but what is happening today promises to be more extensive ( al-Qàdir, 1941; Bouthoul, 1932; Darbshire, 1940; Gauitier, 1924; Guillaume, 1938; Hostelet, 1936; Syrier, 1947).

## Language, knowledge, and power: translation

Understanding the scope of Classical Arab Scholarship is quite essential in locating the Arabic language as a language of scholarship, similar to the way English is established today in the academic world, both through economic and military domination. Sharing parallels with the link between anthropology and colonialism, the classical Arab scholarship has also started with and enabled by a military encounter. Firstly, Alexander’s conquest of Syria (331 BC) has marked translation of Greek texts into Syriac. According to Peter Adamson, by the time we reached 600s, Syriac scholars had excellent access to the works of ancient Greeks ^2^.

Which means, that the initial translation of Greek texts to non-Greek languages did not happen directly from Greek to Arabic. The initial translations to Arabic were from Syriac.

The spread of Arab Conquest, followed by the emergence of Islamic Empires have led to the rise of a new scholarship that is mostly Islamic and almost all in the Arabic language. The significance of politics in the way classical Arab scholarship was shaped was twofold: The master-apprenticeship relationship was quite central in academic training which meant that great scholars had an individual, scholarly, and political value. Secondly, any famous scholar (or I should say, any scholar who started making a name for himself) had to seek the protection of the rich and powerful, who were prepared to spend part of their wealth for the support of science and the arts. In fact, nearly all rulers of the Islamic Empires, including those who are today known as tyrants, seem to have agreed that the patronage of distinctive scholars was one of the obligations of their kingship.

## Ibn Khaldun and *asabiyyah*

Often referred to as the first sociologist, Ibn Khaldun has lived between years 1332 and 1406. He was a jurist and an expert in *fiqh* ( Rosenthal, 1958; Rosenthal, 1983). He can also be seen as a proto ethnographer of the “other” in his work as he wrote the social history of the Berbers ^3^ in North Africa. There are scholars who compared him Max Weber, Adam Smith, Arnold Toynbee, Carl Schmitt, and Niccolo Machiavelli. As an expert in fiqh -the theory and philosophy of the law, Ibn Khaldun systematised the skeleton of thinking in this form of law and legality providing a perfect example to Islamic rationalism ( Amir, 2022) supported by Aristotelian reasoning, somehow closer to today’s secular premises. His life was shaped around movements across the map: he was born in Andalusia and fled to Tunisia. Most of his movements were displacements due to wars taking place in his lifetime. In contemporary world, he is famous for his *Muqaddimah* (1377). *Muqaddimah* (Introduction) was designed to introduce a lengthier book named *Kitāb al-’ihbar* (The Book of Observations)

As it is now known to the scholars of the Middle East and Islam yet less known to anthropologists, his *Muqaddimah*, theorised the rise and falls of human civilizations.

In this work, Ibn Khaldun designed his theory and analysis on the Rise and Fall of empires, under six chapters and I won’t get into the details of the each.

Yet I will use one of the theoretical pillars of Muqaddimah. Its first chapter is an umbrella chapter that explains what he means by human civilization. Then, he divides them into two groups: Desert civilizations, and dynasties.

The cultural evolutionist perspective that we criticize the early anthropology for seems to take a simpler, less categorical, but still hierarchical format here. Ibn Khaldun ranks human societies as primitive and advanced. The fourth chapter is about what happens to civilisations when they stop growing. The last two chapters are on arts and crafts on the one hand, and sciences on the other. These two are also meant to carry suggestions for the civilisations who want to avoid falling to a sedentary state and collapsing, and that is mainly by advancing arts and sciences.

Again, it is important to note that he explains the rise and fall of civilizations through his concept of *asabiyyah*. Often translated as tribalism, the word Asabiyya comes from Asaba, which refers to a group of people who are bound together in a “league of their own.” In brief, he suggests that it is this bond that enables *badawi* groups to be tied via loyalty and succeed. His writing on Assabiya and the dynamics of the rise and fall of dynasties in North Africa has gone on to be enormously influential for the proceeding centuries.

Ibn Khaldun observes *asabiyya* especially in tribal groups with shared religion and value system. Arabs before the big Empires, in easy times of Islam, is the first example he gives to explain asabiyya. But it can be applied to other tribal groups such as Turks, Kurds, and Berber of his time. In his account, this tribal solidarity is the key to explaining both the rise and fall of new political powers. He explains this in a somewhat cyclical manner.

At the beginning of each cycle, a group or tribe achieves military and cultural conquest at the expense of another, fading group. *Asabiyya*, according to Ibn Khaldun, is what gives communities a military advantage in their fighting against sedentary dynasties – *hadara*. They manage this because their *asabiyya* makes such social groups all but irresistible on the battlefield. As the cycle progresses, having achieved victory, the same group then hand on power to the next generation, which consolidates power. Yet in turn once they conquered these dynasties, they became weakened by the seductions of luxury. When a taste of luxury sets in, it leads to inexorable decline. This group becomes the next fading power, ready to be laid low by another tribe, hungry for domination, and inspired by their own group feelings. Adamson refers to this as “A very powerful theory linking culture to “regime change”.” (2016:202)

## A less linear and more circular interaction with “the West”

Ibn Khaldun’s main intervention was to theorize Iberia’s history of the latest 500 years until his time. Before explaining the historical occurrences of the region during those five centuries, I would like to mention the scholars that influenced him.

The connections between the Western scholarship and the Classical Arab scholarship were not a unidirectional one. Meaning, it wasn’t only Ibn Khaldun who influenced Western classical political theories. He himself was influenced by the ancient Greeks. The most important Greek scholar who influenced Ibn Khaldun would be Herodotus. Indeed, Herodotus’ accounts of the Scythians can be seen as a predecessor to accounts of pastoral nomadic tribes. Herodotus also sought to understand the wider importance of cultural and political differences between Greeks and Persians. His writing brings up many of the issues of representation that Edward Said commented on such as his use of the battle between Greeks and Persians to place Greeks, and their alleged love of freedom, in a positive light ( Dewald, 1998) (pages xii-xiii).

Another scholar influenced Ibn Khaldun would be Al-Mas’udi. (Full Name: Abu Hasan ‘Ali ibn al-Husayn al-Mas’udi) He was a historian and geographer. By using both historical and ethnographic material, Al Mas’udi wrote on the Western Middle East and South Asia. Robert Irwin suggests that Al Mas’udi was steeped in the writings of the ancient Greeks in a way that Ibn Khaldun never was. Having said that, he wrote to instruct and to entertain and therefore was not scholarly enough for Ibn Khaldun.

It is important to state that the invasions were often powered by the military strength enabled by strong tribal bonds, asabiyya, in Khaldunian formulation.

It is important to note that, Ibn Khaldun did not design his theory of civilisation to fit a specific historical setting. To make sure his message is understood well, he also explains the fading of the Greeks and Persians, and the original Islamic conquests in the generations after Prophet Mohammad, through *asabiyyah*.

## Founding a discipline

If, as one of the steps towards decolonizing anthropology is to disorient the canon by making non-Western and non-Eurocentric theories and scholars central to the curriculum, then how can we use ibn Khaldun in other anthropology classes as lecturers? It is also suffice to ask, as anthropologists, how can we use Khaldunian theories while, for instance, authoring articles on topics related to politics in the Middle East? I am glad to know that amongst the audience, there are anthropologists who taught Khaldunian theories when they teach topics relevant to his scholarship, such as Middle East cities.

In sum, Ibn Khaldun seem to care for developing a theory that will anatomize the society.

The historical sociology he developed is often found non-normative. Although *Muqaddimah* can be read as a work of political philosophy, it is hard to suggest that he argues for any particular political arrangement. It does not attempt to explain the best way to run a society. Rather, *Muqaddimah* is relentlessly descriptive, with ibn Khaldun occupying the role of the all-seeing, detached observer rather than the role of political advocate. His writing style combined with the novelty of his analysis, has resulted him to be referred as the first sociologist: This makes him different from European scholars like Locke or Hobbes and locates him somewhere closer to Weber. This also explains the repetitive comparisons drawn between these two scholars: Weber and Ibn Khaldun. 

Like Ibn Khaldun himself, his contemporaries were very much aware that he was developing a new discipline. The scholars of his time seem to discuss and treat *asabiyyah* as a new theory, like we do with, for instance, performativity. A term and concept on its own with a theoretical significance.

## Limits and possibilities: thinking anthropologically through Ibn Khaldun

First of all, studying Classical Arab scholars is different than studying classical theorists in contemporary social studies. The main difference is that the scholars who are taught at undergraduate and graduate programmes are already seen and treated as -albeit agreed to be outdated- the fathers of contemporary social theory. There is a vocabulary and an established way of situating the scholars like Thomas Hobbes and John Locke and David Hume. I taught these classical scholars for several years at the university level and the students were already familiar with the conceptual world, even the particulars such as Leviathan and social contract. However, this familiarity is significantly limited when it comes to Classical Arab texts. When it comes to the Arab scholars of classical times, there is not even a consensus about how to transliterate their names into English of into Latin alphabet more broadly. The name of Al-Biruni, the famous polymath and the author of the first ethnographic account on India, for instance, is written in Arabic letters as “البيروني.” Which, then, is transliterated as El-Beruni, Al-Biruni with and without the hyphens (Albiruni), sometimes with accents, as in al-Bīrūnī, and sometimes without the accents. The lack of consensus in Latin spellings of the names of Arab scholars result inconsistencies. Even his country of origin, Khwarezm requires a certain level of familiarity - refers to an area that is today part of Iran and Afghanistan- and the word itself lacks consensus in writing in English with Khwarizm, Khwarazm, Chorasmia and several other variations ^4^.

Those who cannot read classical Arabic will, of course, have to rely on translation, some of which would be outdated. For instance, the English translation copy of Muqaddimah that I have it especially troubling since the translator, Franz Rosenthal, the famous professor of semitic languages at Yale died in 2003, did not include several of the original Arabic words Ibn Khaldun used. This leaving no room for linguistic discussions, which is always important in Arabic texts, and even more so when it comes to certain crucial terms. The core term *asabiyyah*, in the edition I own is repetitively translated it as “group feeling,” and does not enable a conceptual discussion, which is very much present in the original Ibn Khaldun, as seen in the scholarship following him – and citing him.

There are also other limits which require the reader to carry the same filter they use while reading foundational figures in anthropology. Attempting to find and develop universal formulations and deterministic theories, would be the most central of them.

## Limits of Ibn Khaldun as a theorist of colonisation

There are several points to be raised while reading Ibn Khaldun’s theory vis-à-vis current post- colonial theories. One important note to be added is that, in Ibn Khaldun’s formulation, *asabiyya* has its own agency, and is connected to power. I would like to remind that even his description of *asabiyyah* reads very much like a version of “survival of the fittest.”

Luxury is seen as a source of decline and an inability to maintain power. Luxury, in *Muqaddimah*, should not be confused with pleasures. In the parts where Ibn Khaldun discusses luxury, it is more similar to what we can refer to as opulence.

The decline of a civilization then, in Ibn Khaldun’s formulation, is a result of its own weakness, opulence, as the civilization would no longer stay fit and connected. It indulges itself in luxury and opulence.

It is almost as if hegemonic power is formulated uncritically, making “rise” a positive and “fall” a negative signifier, a marker of failure. So it is the right moment I believe to question that, with his uncritical engagement with the workings of power, can we suggest that Ibn Khaldun’s theory had a room for postcolonial critique in the way we think about it today? I think the irony here is quite valuable and is part of the tension I have cherished at the beginning of my paper.

## Ibn Khaldun citation politics

Indeed, we can discuss the limits of Khaldunian theory, yet he still would like to be acknowledged. He says [and I love this part]: “If, I have omitted some point, or if the problems have got confused with something else, the task of correcting remains for the discerning critic; But the merit is mine since I cleared and marked the way” (Ibn Khaldūn, Muqaddimah, pg.42)

Ibn Khaldun seems to be aware of the importance of citation and citation politics as he clearly wants to be cited, like we all do, like Marshall Sahlins does.

## Genealogy and the canon

Before conclusion, I would like to connect a couple of examples where the dismissal to scholars and scholarships of certain periods within the same genealogies create ruptures in our intellectual formations.

To put simply, since all dismissals are selective, they often serve to create and reiterate a particular narrative – a narrative that is politically problematic and scholarly erroneous.

Mainly, we fail to see how the European Enlightenment and the classical Arab thought have been in conversation with one another, complementing and nourishing each other. This mistake is an easy to fall into since there is already an existing popular idea that suggests Islam and the West as cultural opposites of each other. However, as connected geographies across the Mediterranean Sea, the interaction was much more vibrant than a simple opposition of values ^5^.

I observe three main attitudes that I find problematic:

The first one would be to ignore: I have already discussed this through the term “dismissal.” You can call it Eurocentric dismissal.

The second one is to romanticise: “Had the architects of the new political orders in the wake of World Wars I and II been careful readers of Ibn Khaldūn, the rest of twentieth century history might have been very different” (Peter Adamson, Kings College London) Very beautiful but still not helpful.

And there is a third one, which is in immediate conversation with the first category and that is what I call “the Subaltern Backlash” The sort of backlash here at stake is quite assertive with establishing a number of scholarships under the name of Classical Arab scholars and Ibn Khaldun seems to fascinate many. Appropriated to Contemporary Islamist Politics, there are now universities and chairs established and conferences organized under Ibn Khaldun’s name. Although they appear to be part of the decolonizing movement, they are often appropriated by populist right wing Islamist intelligentsia. The main parallel this new subaltern backlash has with the Eurocentric dismissal is to follow the West vs Islam dualism to increase their followership. The problem is, any scholarship written before the colonial encounter is easily accepted as Islamic, including the most secular ones such as Ibn Khaldun’s. While, at the same time, any scholarship and any theory developed after 18 ^th^ century Europe or North America is seen and treated as un-Islamic. One of the most repealing examples they give for un-Islamic theories is Michelle Foucault’s theory of power. Reiterating the West vs Islam dualism allows the local intelligentsia to establish their own canon within the very Eurocentric framework they are expected to be critical about.

Before I move on, I would like to mention that decolonising Islam is yet to be discussed as a political project. That leaves, anti-Western Islamists go unnoticed as products of colonialism. They are the children of colonialism as political projects that perceives Islam as the cultural opposite of the west. Reverse colonialism is still within the conceptual vocabularies of colonialism. This has rich potential for deep and generative discussion, particularly in how it uses the Orientalist projections to specific political aims. If we were to develop the right tools to hang onto to decolonize, it can be applied to each one of these three problematic approaches.

## Locating the critique

I believe critique is not just a charming topic and an “epistemological hypochondria” as once famously put. Critique keeps us on our toes by providing us tools for intellectual progress and advancement.

So at this point, I would like to (be the boring anthropologist and) reflect on the questions of critique and ontology a little bit. There is something deeply concerning when it comes to the question of critique. While on the one hand, a large number of scholars present a convincing argument that “anthropologists (are) best at challenging established ideas and worldviews so as to expand their own” (Eriksen) a number of others express their concerns about the unreliable character of critique as an abstract form of art.

This tension has been witnessed a number of times, but especially in the last five years after French anthropologist Didier Fassin’s keynote address in the annual meeting of the European Association of Social Anthropologists 2016. In this keynote address and in the article published the next year, Fassin presented a sharp defence for critique. He says:

“We must resist both the facile disqualification of critique as a practice passe ´ and the hyperbolic use of critique as a mere mantra, and that anthropology in general and ethnography in particular can help us succeed in this endeavour”

The problem with Fassin’s position was that he was still trying to develop a definition of critique from the canon and within the canon. The pillars of his argument were scholars like Foucault, Bourdieu, and Latour, and Said, all impugned and even slammed the canon of their time. Yet, the critique is a multifaceted process where centralizing the canon works against the very premise of the critique as an attitude. Which is probably why Hage’s intervention to the heated debate around Sahlin and his rant was especially helpful as it was centralizing a critical attitude.

Hage’s suggestion was the following: “To have an anthropological illusio (life pursuit) is also to believe that the labour of disentangling the white from the anthropological/universal is worthwhile. The art of producing a decolonial anthropology is the art of engaging in ethnography while also labouring on this dis-entanglement.”

One of the crucial elements of how to take a critical position would be enabling a conceptually grounded critique – rather than geographically grounded positionalities – and I believe both the Eurocentric dismissal and the populist subaltern backlash are examples to latter. That being, the critical angle needed to expand the conversation and analysis, to question what is today assumed as geographical and cultural other is in fact closer to us and to the formation of our own sense of self (political, cultural, and social self) than we might imagine.

Yet, one of the reasons as to why we need decolonising movement to keep us on our toes is related to the way ontological turn, which is about theorising the local ontologies, is appropriated to the anthropological canon.

A number of scholars have pointed out how the so-called ontological turn runs the risk of deepening and reiterating Eurocentric perspectives and positionalities, no matter how it promises to be established to intervene to it. As we have seen in other turns in anthropology, interventions to the dominant disciplinary tendencies can be appropriated to the very problem they are trying to be formed against, quite rapidly. Instead of introducing a related question to the reader in the conclusion, I instead suggest that even if we embrace the ontological turn uncritically, it is impossible to how can we speak of ontologies without understanding the scholars emerged from that geography who have studied world from their own perspectives?

At this point, I would like to turn one of the applications and studies of Ibn Khaldun that I found to be good example to both decolonising and to rereading classics for an improved genealogy.

The French sociologist and political scientist Hamit Bozarslan, known for his work on state violence and Kurdish struggle, authored an exemplary book. Le lex et la violence (2014) approaches to Ibn Khaldun as a scholar of the state, who is critical to the state’s capabilities, contradictions, and crises. Bozarslan shows us how, according to Ibn Khaldun, state is both the creation and the creator of violence. Contrary to the way contemporary Islamist intelligentsia appropriates Ibn Khaldun, Bozarslan also explains to us how the notion of power is quite central in Khaldunian theory as well. If we read the contemporary Islamist intelligentsia’s books closely, we can easily detect a repeating argument that the theories of power, referring specifically to Foucauldian theory of power, does not exist in “Islamic” thinking, referring to the Classical Arab texts and theories. Bozarslan, on the other hand, helps us trace back to Ibn Khaldun’s very own definition of power ( *mülk*) that is: “[a] general concept designating the exercise of authority through constraint and domination, and as a concrete form of exercise of sovereignty in a determined social and political context.” Admittedly, this definition does not share almost any parallels with Foucauldian definition of power, as he would probably call the Khaldunian definition modernist. Yet, still helps us conclude that the way Ibn Khaldun is made subject to a romantic thinking and the new populisms in the Islamist politics is highly questionable.

## Conclusion: why does including classical Arab thought matter?

When I teach this material to my students, I often provide them examples from the classical texts on what they might assume to be un-Islamic. The one they find the most interesting is usually the theory of evolution, something that can be found in the writings of social scientists, biologists, and also Sufis. An understanding of the classical Arab texts would provide a better understanding of the self and of the other. 

Here, I would like to finish by highlighting that the decolonizing movement’s immediate concern is a pivotal, scholarly speaking: that anthropology as a knowledge-making mechanism should be critical about its engagement with power mechanisms, and the mechanisms that generate inequalities and even violence. At the same time, it questions the way the claims of the decolonizing movement are appropriated to the canon, or even worse, to the operations of various power mechanisms in certain non-Western contexts. Therefore, I also suggested that the main driving concern of the decolonizing movement is, despite the various directions it was interpreted with, is the way it centralizes critique in anthropological and social thought.

## Data Availability

No data are associated with this article.

## Footnotes

^1^To clarify, the initial translation of Greek texts to non-Greek languages did not happen directly from Greek to Arabic. Most of those resources were first translated to another Middle Eastern-East Mediterranean language, Syriac. In around 700s, those Syriac translations started being translated into Arabic and eventually, the direct translations from original Greek sources started emerging as well.

^2^Sadly, most of those translations were lost or destroyed during Mongol invasions between 1207 and 1340.

^3^Although the use of the word Berber is questioned today due to its Roman derogatory reference and Imazighen is instead used to avoid the problem, I will have to follow Ibn Khaldun’s original Arabic text(s) to maintain consistency and surface the critical position I am taking across this text.

^4^Such inconsistencies however do not exist in languages that engage with this scholarship more closely, such as Persian and Turkish

^5^I am not even getting into how west vs Islam are categorically incorrect dualities.
