# Genealogy, critique, and decolonisation: Ibn Khaldun and moving beyond filling the gaps

**DOI:** 10.12688/openreseurope.16148.2

**Published:** 2025-05-16

**Authors:** Sertaç Sehlikoglu

**Affiliations:** 1 Institute for Global Prosperity, University College London, London, England, W1T 7NF, UK

**Keywords:** Decolonising, Anthropology Theory, Genealogy, Critique

## Abstract

The aim of this paper is to locate critique at the intersections of the genealogy of knowledge in anthropological thinking and the decolonising movement. The paper approaches the decolonising movement as one of the most crucial points in anthropological thinking. It is built on the premise that the decolonising movement is set to go beyond filling the gaps in genealogies and it can do so by: (1) revising the ‘dismissed’ genealogies that have contributed to the formation of the contemporary classical theory and (2) thinking creatively in implementing the critical thinking tools to the dismissed scholarship, in an equal manner to the Eurocentric scholarship. To illustrate, it uses the case of Ibn Khaldun, an Arab scholar of social sciences and historical analysis from the 14 ^th^ Century, often referred to as the first sociologist. On the one hand, his influence on classical Western thinking is largely dismissed. On the other hand, as a counter-response to this dismissal, the new Islamic revivalist intelligentsia in the Muslim right engages with him in a selective manner that not only rejects that central critical thinking but, even worse, sanctions the local regimes of power, including that local canon. By locating his scholarship to multiple tropes in anthropological theory and reading his evolutionist thinking vis-à-vis the post-colonial literature in anthropology and sociology, I question the limits and possibilities of critical thinking within and beyond the decolonising movement.

## Introduction

The aim of this paper is to locate critique at the intersections of the genealogy of knowledge in anthropological thinking and the contemporary efforts of the decolonising movement. I connect a two-tiered set of questions as a way to inquire about the limits and possibilities of critical thinking within and beyond the decolonising movement.

In the first tier, the paper criticizes the series of work in anthropological theory that tends somehow to start their genealogies with the Ancient Greek thinkers and then jump for about two millennia and continue with enlightenment, omitting all the debates, conversations, and disagreements (the three main components of the evolution of social thought) that took place in between. This leap, in other words, involves a dismissal of the series of scholarly conversations and advancements that took place from 400 BC to the 1500s across the Mediterranean Sea. Then, in this first layer, I engage with the interconnected questions of intellectual impoverishment due to omission and questions on the method of engagement. I, therefore, bring in one of the many dismissed scholarships with crucial value to the genealogy of knowledge we today claim as Western: the Classical Arab Scholarship. By Classical Arab Scholarship, I refer to the substantial number of works written in Arabic and circulated across North Africa, the Middle East, and parts of East Asia from roughly the late 900s to early 1400s. The word Arab in this category of Classical Arab Scholarship does not refer to the ethnic background of the scholars, nor does it necessarily signify any particular religion. Rather, it is used to refer to the language in which the individuals from various ethnic backgrounds were trained and had authored their work. In this first layer, I ask: What happens when a massive chunk of rich scholarly developments and conversations, which took place for as long as 700 or so years, is removed from our sense of literature, our readings of the resources, including both the classical and ancient texts? What does it mean to have interruptions in this genealogy? What epistemological scope does this selective reading and referencing bring to our understanding of knowledge? Should we address such substantial gaps and, if so, how should we do that? I am not answering each one of those questions in this paper but laying them out to warm up. In this first tier, I approach classical Arab scholarship as both Western and non-Western at the same time.

In the second tier, I then question the “how” of the decolonising curriculum. My main question there would be whether and how we are to engage with the native scholars in anthropology and with their theories. Bear in mind that some of those theories were part of the genealogical knowledge productions but were today dismissed. So my inquiry then inevitably has another lingering effect: is filling the gaps enough to decolonise? To explore this second point more deeply, I examine Ibn Khaldun's theory of state formation and power relations as a key example from classical Arab scholarship.

This analysis builds on recent scholarship examining the integration of non-Western theoretical frameworks into anthropological thinking ( Hage, 2017; Harrison, 1991; MacClancy & Bennett, 2002). By examining Ibn Khaldun's contributions, this paper contributes to ongoing debates about how decolonial approaches can move beyond simply adding diverse voices to existing canons. I critically engage with the ways in which he is appropriated by various local regimes of power in the Middle East and various Western romantic views.

The topic “decolonising movement” is highly contested and thus, it is important clarify from the beginning that this paper does not claim a universal truth or principle about *how* to decolonise. What it does very clearly, however, is take a denunciatory position against the contexts where the decolonising movement is appropriated in non-Western contexts to silence the critical voices. Therefore, the paper also touches on the cases where the new Islamic revivalist intelligentsia in the Muslim right engage with, praised, and even used Ibn Khaldun in a selective manner that paradoxically both: a) establishes a canon voice that marginalises critical voices and b) directly or indirectly serves to the local regimes of power.

In conclusion, I complete my circular thinking by returning to the question of “dismissal” as a failure of scholarly diligence and intellectual imagination.

## Selected debates on decolonising anthropology

It would be helpful to start by reflecting on what it means to decolonise knowledge in general and decolonise anthropology in particular.

With the help of Foucauldian theory on power and knowledge, anthropological knowledge has been criticized, especially in the ‘80s and ‘90s, as a discipline that emerged from Western colonization and as a result of Western colonization. The significance of the postmodern turn in anthropology was that it has stimulated a wave of thinking that questions the legitimacy of various sciences and social sciences as knowledge-making mechanisms.

For the sake of staying on the nuanced side of the conversation and of the probe, it is important to remind ourselves of one more concern in the formation of anthropology as a discipline. Anthropology as a discipline was initially established to form scientific knowledge about the colonially encountered others (and, of course, through others about themselves). Since a large number of early anthropologists were financially supported by the colonial officers (if not acting as one), during the periods preceding the establishment of anthropology as a discipline, the knowledge produced about this encountered “other” was through the travel notes of the diplomats.

A considerable number of those popular works were about places they had never been to and the people they had never met. Just to give one single example, Richard Knolles published his book “General History of the Turks” in 1603, without a single visit to the geography he wrote about. The book became so popular that it had seven editions ( Ingram, 2015).

Establishing a discipline that will use scientific standards of knowledge production to study the newly encountered societies was, therefore, one of the rationales behind the need for a discipline like anthropology. And here is the crucial point:

What is considered ‘scientific’ has not been proven to provide a reliable ground for this new discipline. The scientific requirements to be met at the time were highly problematic. Namely, cultural evolutionism and the preoccupation for cataloging various races to fit into this unilinear sense of social progress embedded in cultural evolutionism had later become abandoned practices and methods specifically for promoting what we today call scientific racism. Written when cultural evolutionism was considered to be an accepted fact, the language used in early anthropological texts assumed racial, social, and intellectual superiority of the industrialized societies and, thus, referred to Aboriginal and Indigenous groups as *primitives* and even *savages*.

From the perspective of the decolonising movement, therefore, the issue at stake was more than having racist undertones that “could” be discussed whether they were actually racist or not. The issue at stake is how several of those works have actively contributed to the production of knowledge that suggests and even ranks racial hierarchies.

The postcolonial theory has challenged the scholarly legitimacy of these early works. The decolonising movement takes a more radical position, however. The decolonising movement has emerged out of disappointment and frustration with the way the curriculums are formed, and citation politics are shaped in a manner that still does not address the native scholars’ critical interventions. The dismissal of critical interventions meant attempting to continue canonical writing as if, for instance, Frantz Omar Fanon never reminded us of the centrality of race and Eurocentrism in psychoanalysis and studies on self ( Fanon, 1961; Fanon, 1963), Edward Said (1978) never wrote hundreds of pages to demonstrate English literature is not solely English, Gayatri Spivak ( 1988; 1996) has not introduced the terms essentialism and strategic essentialism to our vocabularies. The very dismissal, therefore, has simultaneously resulted in postcolonial theory being treated as ‘fads’ to be moved on and moved away from. As a response to this broader problem of citation politics and canonical writing, the decolonising movement has emerged believing that a more fundamental position is required, by replacing the existing dismissive canon with the scholarship of the marginalized native scholars.

While those who supported and joined the decolonising movement were taking issues with the anthropological canon for being dismissive to the native scholars, the older generation of scholars who form the anthropological canon had another concern. Their concern was about how to keep the anthropological canon in the canon. But then, how would we separate what is anthropological apart from what is not?

On 31 ^st^ of August in 2017, the renowned anthropologist Marshall Sahlins published a blurb on the HAU Facebook page where he asked “Where Have All the Cultures Gone?”. He starts by asking: “What happened to anthropology as encompassing human science? Why is a century of the first- hand ethnography of cultural diversity now ignored in the training and work of anthropologists?” He questions the way contemporary anthropology has started dismissing its own foundational knowledge.

Sahlins then continues by listing several examples that he believes to be removed from the recent anthropology curriculum including Naga head-hunting, Fijian cannibalism, Amazonian animism, and Aztec human sacrifice. All of these topics are those I teach and show images with viewer discretion and a courtesy warning, in line with contemporary pedagogies sensitive to diversity and inclusivity. What Sahlin suggests, however, is that anthropologists are “the custodians of this knowledge, and we are content to let it be forgotten.” With his social media ranting, Sahlins sparked a heated debate within anthropological circles, which was quite exciting for a number of us.

Sahlin’s position was the one shared by many traditional anthropologists, especially in the UK but also in the US and Europe. That, all the critical movements and conceptual turns, and waves in anthropology have actually hijacked what anthropology should be about. The tension seems to be about the very issue of what anthropology is and should be about. The historical foundation of anthropology as a discipline has shaped both the definition of and critical interventions to anthropology and anthropological thinking. Yet the issue of critique and genealogy rarely goes well with what this paper keeps referring to as dismissal.

It is important to clarify the tension that, the anthropology Sahlins refers to is a foundational anthropology that current theories were built onto and are thus no longer considered advanced enough to use in understanding today’s world. Yet, it is equally important to keep the progressive evolutionist understanding in knowledge-making processes under probe. That, our contemporary knowledge is established through the past ones that were not advanced enough, but still have been part of the production processes of today’s what we perceive to be more advanced theories and frameworks.

This paper accepts the central tension in this debate on *genealogy* and the gaps as productive ones. It will apply this idea to the decolonising curriculum in anthropology by using the case of Ibn Khaldun. I join post-colonial theorist Ghassan Hage, who, while responding to the conversation in the aftermath of Sahlin’s self-declared rant, said that there are two kinds of critique. While some are dominating, silencing, and therefore *paralyzing*, some other critiques are continued intellectual discussions, open up new venues, and therefore are *enabling*. In this piece, he called the anthropologists who are involved in the decolonising movement to do a more productive and enabling critique of carefully dismantling the problematic elements while staying in conversation with the former generation of scholars. In other words, in Hage’s account, the decolonising movement is about critiquing the canon but, while doing so, creating a more thorough, detailed, stronger and comprehensive genealogy of knowledge.

## Classical Arab scholarship

**Figure 1.**
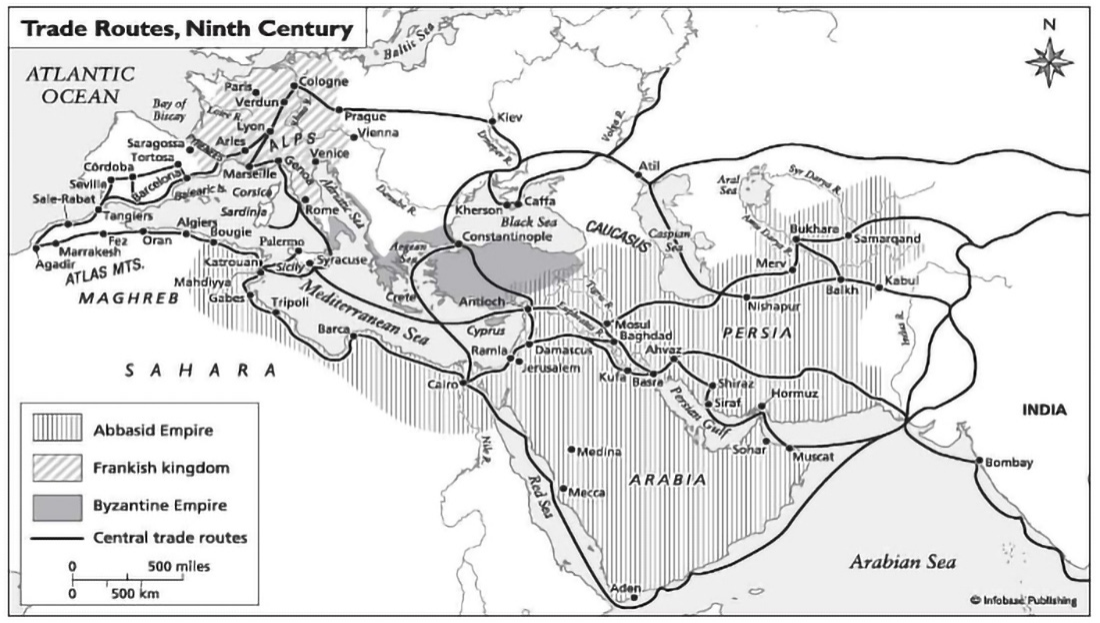
Trade Routes that also shaped the transfer of books and scholarship. Source: http://www.worldhistory.biz/sundries/32597- trade-and-agriculture-under-the-abbasids.html.

If we turn to Classical Arab texts at this point of conversation, we need to remind ourselves that they offer more than simple alternative forms of knowledge. It is important to understand the Classical Arab scholarship as a vast and diverse number of theories and debates that were produced at dozens of institutions connected to each other via trade routes across the Mediterranean and expanding to Central Asia ( Figure 1), which translated and furthered the ancient Greek knowledge of philosophy and sciences. A number of works from this scholarship were later on translated into European languages during the Enlightenment.

We should take a moment and repeat the earlier question on why we, anthropologists and social scientists, find it acceptable, if not exciting, to use theories of the ancient Greeks but not the classical Arab scholars. Different approaches suggest different durations, although for at least seven hundred years the ancient Greeks’ work in philosophy, biology, and mathematics were translated, studied and, most importantly, further developed by the Arab scholars ^1^. Surely, in a duration of 700 years, there must be some scholarly work worthy of incorporating into our thinking, of engaging with, of giving reference to (even just to disagree)? Why do we skip those works altogether? And more importantly, what analytical limits do we establish through such gaps in the genealogy of knowledge?

Recent scholarship has demonstrated the extensive influence of Classical Arab thought on European intellectual traditions (add citations). For example, studies have traced how Ibn Khaldun's theories of social solidarity influenced early European social theory through translations and scholarly networks (add specific citation). This evidence challenges simplistic divisions between 'Western' and 'Islamic' intellectual traditions.

A similar rise in interest took place in the 1930s and 1940s mostly in French-speaking academia ( Bouthoul, 1932; Darbishire, 1940; Hostelet, 1936; Qadir, 1941; Syrier, 1947), but what is happening today promises to be more extensive. In fact, today we witness an exciting moment. We are witnessing a moment with an incredible rise in applying these Classical Arab scholars, such as Ibn Khaldun, al-Ghazali, Ibn Arabi, and Albiruni to core theoretical analysis in the English and French-speaking academia. Contemporary interests include Alireza Doostdar’s work where he ( 2018) “The Iranian Metaphysicals” where he successfully examines uncertainties about the supernatural among the urban middle class groups/individuals in Tehran, Iran. He carefully explains us the connections between the classical metaphysicals’ theories and the modern scientific reason’s empirical power.

Language, knowledge, and power: translation Understanding the scope of Classical Arab Scholarship is quite essential in locating the Arabic language as a language of scholarship, similar to the way English is established today in the academic world, both through economic and military domination. Sharing parallels with the link between anthropology and colonialism, the classical Arab scholarship also started with and was enabled by a military encounter. Firstly, Alexander’s conquest of Syria (331 BC) marked the translation of Greek texts into Syriac. According to Peter Adamson, by the time we reached 600s, Syriac scholars had excellent access to the works of ancient Greeks ^2^.

Which means, that the initial translation of Greek texts to non-Greek languages did not happen directly from Greek to Arabic. The initial translations to Arabic were from Syriac.

The spread of Arab conquest and subsequent emergence of Islamic Empires fostered the development of a new intellectual tradition that, while predominantly Islamic in character, was conducted almost entirely in Arabic. This scholarship emerged through a complex system of political and institutional relationships that shaped its development in two crucial ways.

First, knowledge transmission operated through a formalized master-apprenticeship system. This arrangement meant that distinguished scholars possessed not only intellectual authority but also significant political influence, as their ability to train future scholars gave them considerable social power.

Second, the system depended heavily on patronage networks. Scholars who gained recognition needed to secure the protection and support of wealthy and powerful patrons who would fund their scientific and artistic pursuits. This patronage system became so deeply embedded in Islamic political culture that even rulers known today for their tyrannical tendencies viewed the support of distinguished scholars as a fundamental obligation of kingship.

This system of scholarship operated within an extensive network of intellectual exchange spanning multiple centuries and regions. It was characterized by several key features: systematic translation movements that not only preserved but expanded upon Greek philosophical and scientific works; the establishment of major learning centers stretching from Baghdad to Córdoba; sophisticated systems of scholarly training and knowledge transmission; and complex patronage relationships that simultaneously enabled and constrained intellectual production.

Understanding these historical processes provides crucial insight into how Classical Arab scholarship developed as both an influence on and product of broader intellectual traditions. The interplay between political patronage and scholarly independence created a dynamic intellectual culture that would have a lasting impact on the development of knowledge across the medieval world.

## Ibn Khaldun and *asabiyyah*

Often referred to as the first sociologist, Ibn Khaldun lived between years 1332 and 1406. He was a jurist and an expert in *fiqh* ( Rosenthal, 1958; Rosenthal, 1983). He can also be seen as a proto-ethnographer of the “other” in his work as he wrote the social history of the Berbers ^3^ in North Africa. Some scholars compared him to Max Weber, Adam Smith, Arnold Toynbee, Carl Schmitt, and Niccolo Machiavelli. As an expert in *fiqh* -the theory and philosophy of the law, Ibn Khaldun systematized the skeleton of thinking in this form of law and legality providing a perfect example to Islamic rationalism ( Amir, 2022) supported by Aristotelian reasoning, somehow closer to today’s secular premises. His life was shaped around movements across the map: he was born in Andalusia and fled to Tunisia. Most of his movements were displaced due to wars taking place in his lifetime. In contemporary world, he is famous for his *Muqaddimah* (1377). *Muqaddimah* (Introduction) was designed to introduce a lengthier book named *Kitāb al-’ihbar* (The Book of Observations)

As it is now known to the scholars of the Middle East and Islam yet less known to anthropologists, his *Muqaddimah* theorized the rise and fall of human civilizations.

In this work, Ibn Khaldun designed his theory and analysis of the Rise and Fall of empires under six chapters, and I won’t get into the details of each.

Yet I will use one of the theoretical pillars of Muqaddimah. Its first chapter is an umbrella chapter that explains what he means by human civilization. Then, he divides them into two groups: Desert civilizations and dynasties.

The cultural evolutionist perspective that we criticize early anthropology for seems to take a simpler, less categorical, but still hierarchical format here. Ibn Khaldun ranks human societies as primitive and advanced. The fourth chapter is about what happens to civilizations when they stop growing. The last two chapters are on arts and crafts on the one hand and sciences on the other. These two are also meant to carry suggestions for the civilizations who want to avoid falling into a sedentary state and collapsing, and that is mainly by advancing arts and sciences.

Again, it is important to note that he explains the rise and fall of civilizations through his concept of *asabiyyah*. The concept of *asabiyyah*, while often simplified as 'group feeling' or 'tribalism', represents a sophisticated theory of social cohesion and political power. Ibn Khaldun uses this concept to explain both micro-level social bonds and macro-level political transformations, anticipating later sociological theories of social capital and political legitimacy ( Alatas, 2014; Çelebi, 2010; Hassan, 1977). His analysis of how *asabiyyah* functions in different social contexts demonstrates an early form of comparative political analysis that remains relevant to contemporary social theory.

Ibn Khaldun observes *asabiyya*, especially in tribal groups with shared religion and value system. Arabs before the big Empires, in the easy times of Islam, is the first example he gives to explain asabiyya. But it can be applied to other tribal groups such as Turks, Kurds, and Berber of his time. In his account, this tribal solidarity is the key to explaining both the rise and fall of new political powers. He explains this in a somewhat cyclical manner.

At the beginning of each cycle, a group or tribe achieves military and cultural conquest at the expense of another fading group. *Asabiyya*, according to Ibn Khaldun, is what gives communities a military advantage in their fighting against sedentary dynasties – *hadara*. They manage this because their *asabiyya* makes such social groups all but irresistible on the battlefield. As the cycle progresses, having achieved victory, the same group then hand on power to the next generation, which consolidates power. Yet in turn once they conquered these dynasties, they became weakened by the seductions of luxury. When a taste of luxury sets in, it leads to inexorable decline. This group becomes the next fading power, ready to be laid low by another tribe, hungry for domination, and inspired by their own group feelings. Adamson refers to this as “A very powerful theory linking culture to “regime change”.” (2016:202)

## A less linear and more circular interaction with “the West”

Ibn Khaldun’s main intervention was to theorize Iberia’s history of the latest 500 years until his time. Before explaining the historical occurrences of the region during those five centuries, I would like to mention the scholars who influenced him.

The connections between the Western scholarship and the Classical Arab scholarship were not unidirectional. Means that it wasn’t only Ibn Khaldun who influenced Western classical political theories. He himself was influenced by the ancient Greeks. The most important Greek scholar who influenced Ibn Khaldun would be Herodotus. Indeed, Herodotus’ accounts of the Scythians can be seen as a predecessor to accounts of pastoral nomadic tribes. Herodotus also sought to understand the wider importance of cultural and political differences between Greeks and Persians. His writing brings up many of the issues of representation that Edward Said commented on such as his use of the battle between Greeks and Persians to place Greeks, and their alleged love of freedom, in a positive light ( Dewald, 1998) (pages xii–xiii).

Another scholar who influenced Ibn Khaldun was Al-Mas’udi. (Full Name: Abu Hasan ‘Ali ibn al-Husayn al-Mas’udi) He was a historian and geographer. By using both historical and ethnographic material, Al Mas’udi wrote on the Western Middle East and South Asia. Robert Irwin (2018) suggests that Al Mas’udi was steeped in the writings of the ancient Greeks in a way that Ibn Khaldun never was. Having said that, he wrote to instruct and to entertain and therefore was not scholarly enough for Ibn Khaldun.

It is important to state that the invasions were often powered by the military strength enabled by strong tribal bonds, asabiyya, in Khaldunian formulation.

It is important to note that Ibn Khaldun did not design his theory of civilization to fit a specific historical setting. To ensure his message is understood well, he also explains the fading of the Greeks and Persians and the original Islamic conquests in the generations after Prophet Mohammad through *asabiyyah*.

## Founding a discipline

Suppose one of the steps towards decolonising anthropology is to disorient the canon by making non-Western and non-Eurocentric theories and scholars central to the curriculum. How can we use ibn Khaldun in other anthropology classes as lecturers? It is also sufficient to ask, as anthropologists, how we can use Khaldunian theories while, for instance, authoring articles on topics related to politics in the Middle East. There are several anthropologists who taught Khaldunian theories when they teach topics relevant to his scholarship, such as Middle East cities, including Alice Wilson (Sussex)and in broader social theory Ross Higgins (Concordia).

In sum, Ibn Khaldun seems to care for developing a theory that will anatomize society.

The historical sociology he developed is often found to be non-normative. Although *Muqaddimah* can be read as a work of political philosophy, it is hard to suggest that he argues for any particular political arrangement. It does not attempt to explain the best way to run a society. Rather, *Muqaddimah* is relentlessly descriptive, with Ibn Khaldun occupying the role of the all-seeing, detached observer rather than the role of political advocate. His writing style, combined with the novelty of his analysis, has resulted in him being referred to as the first sociologist. This makes him different from European scholars like Locke or Hobbes and locates him somewhere closer to Weber. This also explains the repetitive comparisons drawn between these two scholars: Weber and Ibn Khaldun.

Like Ibn Khaldun himself, his contemporaries were very much aware that he was developing a new discipline. The scholars of his time seem to discuss and treat *asabiyyah* as a new theory, like we do with, for instance, performativity. A term and concept on its own with a theoretical significance.

Limits and possibilities: thinking anthropologically through Ibn Khaldun Studying Classical Arab scholars is different from studying classical theorists in contemporary social studies. The main difference is that the scholars who are taught at undergraduate and graduate programmes are already seen and treated as, albeit agreed to be outdated, the fathers of contemporary social theory. Thinkers like Hobbes, Locke, and Hume come with a built-in conceptual vocabulary familiar to most students, reinforced through curricula and citation traditions. I taught these classical scholars for several years at the university level and the students were already familiar with the conceptual world, even the particulars such as Leviathan and social contract. In contrast, scholars like Ibn Khaldun exist at the margins of these canons, making their integration both theoretically enriching and pedagogically demanding. The complexity of engaging with Classical Arab texts extends beyond theoretical challenges to practical issues of translation and interpretation. These technical challenges, while important to acknowledge, should not detract from the broader theoretical contributions these texts offer to contemporary anthropological thought.

Part of this challenge lies in technical issues such as transliteration and translation that even impacts the variety of names used to refer to the most significant figures. The name of Al-Biruni, the famous polymath and the author of the first ethnographic account on India, for instance, is written in Arabic letters as “البيروني.” Which, then, is transliterated as El-Beruni, Al-Biruni with and without the hyphens (Albiruni), sometimes with accents, as in al-Bīrūnī, and sometimes without the accents. The lack of consensus in Latin spellings of the names of Arab scholars result inconsistencies. Even his country of origin, Khwarezm requires a certain level of familiarity - refers to an area that is today part of Iran and Afghanistan- and the word itself lacks consensus in writing in English with Khwarizm, Khwarazm, Chorasmia and several other variations ^4^.

Those who cannot read classical Arabic will, of course, have to rely on translation, some of which would be outdated. For instance, the English translation copy of Muqaddimah that I have it especially troubling since the translator, Franz Rosenthal, the famous professor of semitic languages at Yale died in 2003, did not include several of the original Arabic words Ibn Khaldun used. This leaves no room for linguistic discussions, which is always important in Arabic texts, and even more so when it comes to certain crucial terms. The core term *asabiyyah*, in the edition I own, is repetitively translated as “group feeling,” and does not enable a conceptual discussion, which is very much present in the original Ibn Khaldun, as seen in the scholarship following him – and citing him.

There are also other limits which require the reader to carry the same filter they use while reading foundational figures in anthropology. Attempting to find and develop universal formulations and deterministic theories, would be the most central of them.

## Limits of Ibn Khaldun as a theorist of colonisation

When read alongside postcolonial theory, Ibn Khaldun’s work reveals both conceptual value and notable limitations. His understanding of *asabiyyah* as an autonomous force of political cohesion offers a lens into premodern state formation and social solidarity. Yet his formulation often echoes survivalist logics — power is gained and lost through strength, and luxury is viewed as a harbinger of decline. Luxury is seen as a source of decline and an inability to maintain power. Luxury, in *Muqaddimah*, should not be confused with pleasures. In the parts where Ibn Khaldun discusses luxury, it is more similar to what we can refer to as opulence.

In this context, “rise” and “fall” (of the civilisations) are not neutral descriptors but moralised outcomes, where ascendency suggests virtue and decadence implies failure. There is little interrogation of power itself — its violence, exclusion, or ideological machinery. This absence raises a crucial question: can Ibn Khaldun’s theory accommodate a postcolonial critique of power and representation? Likely not in full. But that tension, rather than discrediting his thought, invites us to read him critically and generatively — to explore what his framework makes visible and what it obscures. The irony here is quite valuable and is part of the tension cherished at the beginning of this paper.

This question reveals a key tension in decolonial approaches to classical texts. While Ibn Khaldun's work precedes modern colonialism, his power and social change theories contain insights and limitations for contemporary decolonial theory. His analysis of how political power operates through social bonds and cultural practices anticipates some aspects of postcolonial theory, yet his relatively uncritical approach to power hierarchies reflects the limitations of his historical context. Recent scholars have shown how reading Ibn Khaldun alongside postcolonial theory can generate new insights about both historical and contemporary power relations ( Amir, 2022; Bozarslan, 2014; Tabatabai, 2024).

## Genealogy, citation, and the canon

Indeed, we can discuss the limits of Khaldunian theory, yet he still would like to be acknowledged, read, and cited. As he writes in Muqaddimah: “If I have omitted some point, or if the problems have got confused with something else, the task of correcting remains for the discerning critic; but the merit is mine since I cleared and marked the way.” (pg 42) Ibn Khaldun seems to be aware of the importance of citation and citation politics as he clearly wants to be cited, like we all do, like Marshall Sahlins did during his lifetime. This plea for intellectual recognition underscores the politics of citation that shape not only our readings but also our exclusions.

Dismissals — whether through Eurocentrism or ideological backlash — fracture our genealogies of knowledge. We often overlook the profound entanglements between Classical Arab thought and European Enlightenment, defaulting instead to simplified oppositions between Islam and the West. These simplifications enable three problematic narratives. The first one is the Eurocentric dismissal of non-Western thought, as critiqued in this paper and in a range of decolonial scholarship. Second is the romanticisation of premodern scholars as ideal solutions ( Adamson, 2016). I find the romantic fascination quite beautiful, but often unhelpful. Third is the reactionary backlash that weaponises Ibn Khaldun and the legacy of CAS for nationalist projects; I call this “the Subaltern Backlash”. The sort of backlash here at stake is quite assertive with establishing a number of scholarships under the name of Classical Arab scholars, and Ibn Khaldun seems to fascinate many ( Alatas, 2014; Amir, 2022; Han, 2022; Kayapınar, 2019). Appropriated to Contemporary Islamist Politics, universities and chairs are now established and conferences organized under Ibn Khaldun’s name. Although they appear to be part of the decolonising movement, they are often appropriated by populist right-wing Islamist intelligentsia and attempt to canonize the anti-Western politics.

The main parallel this new subaltern backlash has with the Eurocentric dismissal is to follow the West vs Islam dualism to increase their followership ( Iqtidar, 2011; Iqtidar, 2024; Sehlikoglu & Kurt, 2024). The problem is that any scholarship written before the colonial encounter is easily accepted as Islamic, including the most secular ones such as Ibn Khaldun’s. At the same time, any scholarship and any theory developed after 18 ^th^ century Europe or North America is seen and treated as un-Islamic. One of the most appealing examples they give for un-Islamic theories is Michelle Foucault’s theory of power ( Alatas, 2014). Reiterating the West vs Islam dualism allows the local intelligentsia to establish their own canon within the very Eurocentric framework they are expected to be critical about ( Sehlikoglu *et al.*, 2025).

Today, Ibn Khaldun’s legacy illustrates all three. He is ignored by many, overly idealised or romanticised by some, and appropriated by others to construct a counter-canon that reproduces the very binaries it seeks to dismantle. Decolonisation, if it is to remain intellectually honest, must resist all three moves. It must acknowledge that Ibn Khaldun’s work — like any theoretical tradition — contains insights, omissions, and contradictions. Our task is not to canonize him, but to situate him: critically, carefully, and contextually.

Further, we often fail to see how the European Enlightenment and the classical Arab thought have been in conversation with one another, complementing and nourishing each other. This mistake is an easy to fall into since there is already an existing popular idea that suggests Islam and the West as cultural opposites of each other. However, as connected geographies across the Mediterranean Sea, the interaction was much more vibrant than a simple opposition of values ^5^.

Before I move on, I would like to mention that decolonising Islam is yet to be discussed as a political project. That leaves anti-Western Islamists to go unnoticed as products of colonialism. They are the children of colonialism as political projects that perceive Islam as the cultural opposite of the West. Reverse colonialism is still within the conceptual vocabularies of colonialism. This has rich potential for deep and generative discussion, particularly in how it uses Orientalist projections for specific political aims. If we were to develop the right tools to hang onto to decolonise, it could be applied to each one of these three problematic approaches.

## Locating the critique

I believe critique is not just a charming topic and an “epistemological hypochondria” as once famously put. Critique keeps us on our toes by providing us tools for intellectual progress and advancement.

So at this point, I would like to (be the boring anthropologist and) reflect on the questions of critique and ontology a little bit. There is something deeply concerning when it comes to the question of critique. While on the one hand, a large number of scholars present a convincing argument that “anthropologists (are) best at challenging established ideas and worldviews so as to expand their own” (Eriksen) a number of others express their concerns about the unreliable character of critique as an abstract form of art.

This tension has been witnessed a number of times, but especially in the last five years after French anthropologist Didier Fassin’s keynote address in the annual meeting of the European Association of Social Anthropologists 2016. In this keynote address and in the article published the next year, Fassin presented a sharp defense for critique. He says:

“We must resist both the facile disqualification of critique as a practice passe and the hyperbolic use of critique as a mere mantra, and that anthropology in general and ethnography in particular can help us succeed in this endeavour”

The problem with Fassin’s position was that he was still trying to develop a definition of critique from the canon and within the canon. The pillars of his argument were scholars like Foucault, Bourdieu, and Latour, and Said, all impugned and even slammed the canon of their time. Yet, the critique is a multifaceted process where centralizing the canon works against the very premise of the critique as an attitude. This is probably why Hage’s intervention in the heated debate around Sahlin and his rant was especially helpful, as it was centralized a critical attitude.

Hage suggested the following: “To have an anthropological illusio (life pursuit) is also to believe that the labour of disentangling the white from the anthropological/universal is worthwhile. The art of producing a decolonial anthropology is the art of engaging in ethnography while also labouring on this dis-entanglement.”

One of the crucial elements of how to take a critical position would be enabling a conceptually grounded critique – rather than geographically grounded positionalities – and I believe both the Eurocentric dismissal and the populist subaltern backlash are examples to latter. That being, the critical angle needed to expand the conversation and analysis, to question what is today assumed as geographical and cultural other is, in fact, closer to us and to the formation of our own sense of self (political, cultural, and social self) than we might imagine.

Yet, one of the reasons as to why we need decolonising movement to keep us on our toes is related to the way ontological turn, which is about theorizing the local ontologies, is appropriated to the anthropological canon.

A number of scholars have pointed out how the so-called ontological turn runs the risk of deepening and reiterating Eurocentric perspectives and positionalities, no matter how it promises to be established to intervene to it. As we have seen in other turns in anthropology, interventions to the dominant disciplinary tendencies can be appropriated to the very problem they are trying to be formed against, quite rapidly. Instead of introducing a related question to the reader in the conclusion, I instead suggest that even if we embrace the ontological turn uncritically, it is impossible to how can we speak of ontologies without understanding the scholars emerged from that geography who have studied world from their own perspectives?

At this point, I would like to turn one of the applications and studies of Ibn Khaldun that I found to be good example to both decolonising and to rereading classics for an improved genealogy.

The French sociologist and political scientist Hamit Bozarslan, known for his work on state violence and the Kurdish struggle, authored an exemplary book. Bozarslan (2014) approaches to Ibn Khaldun as a scholar of the state, who is critical to the state’s capabilities, contradictions, and crises. Bozarslan shows us how, according to Ibn Khaldun, state is both the creation and the creator of violence. Contrary to the way contemporary Islamist intelligentsia appropriates Ibn Khaldun, Bozarslan also explains to us how the notion of power is quite central in Khaldunian theory as well. Let's read the contemporary Islamist intelligentsia’s books closely. We can easily detect a repeating argument that the theories of power, referring specifically to Foucauldian theory of power, do not exist in “Islamic” thinking, referring to the Classical Arab texts and theories. Bozarslan, on the other hand, helps us trace back to Ibn Khaldun’s very own definition of power ( *mülk*) that is: “[a] general concept designating the exercise of authority through constraint and domination, and as a concrete form of exercise of sovereignty in a determined social and political context.” Admittedly, this definition does not share almost any parallels with Foucauldian definition of power, as he would probably call the Khaldunian definition modernist. Yet, it still helps us conclude that the way Ibn Khaldun is made subject to romantic thinking and the new populisms in Islamist politics is highly questionable.

## Conclusion: why does including classical Arab thought matter?

When teaching classical Arab scholarship as part of decolonising curriculum, I challenge students' preconceptions about what is "Islamic" versus "un-Islamic" - binary oppositions that often reflect post-colonial conditioning rather than historical reality. A powerful example is the theory of evolution, which many assume to be inherently un-Islamic, yet appears throughout classical Arab texts and endorsed by biologists, philosophers, Sufis, medical scholars, and other polymaths. Ibn Khaldun's Muqaddimah presents a remarkably evolutionary framework, describing progression from minerals to plants to animals and finally humans, even noting connections between advanced primates and early humans. This perspective wasn't unique to Ibn Khaldun; Ibn Arabi similarly articulated evolutionary concepts as part of divine creation rather than opposed to it.

These examples illustrate that incorporating classical Arab scholarship into contemporary theory isn't merely about filling gaps in our intellectual genealogies - it fundamentally transforms our understanding of both self and other. This recovery work supports the decolonising movement's core concern that anthropology must critically examine its entanglement with power systems that generate inequalities. However, we must remain vigilant against the appropriation of decolonising concepts, whether to serve Western academic canons or to reinforce power structures in non-Western contexts. This ongoing vigilance underscores why critique must remain central to anthropological thought, continuously questioning both our objects of study and our frameworks of understanding.

I take issues what you may call "genealogical fantasy" - the idea that contemporary Western thought emerged directly from ancient Greek philosophy, skipping over the crucial intermediary role of Arab and Islamic scholarship during the medieval period. This common narrative creates an artificial gap of nearly a millennium in intellectual history. This imagined genealogy erases how classical Arab scholars like Ibn Khaldun (who developed sophisticated theories of social cohesion and historical change), Al-Biruni (who conducted methodical cross-cultural studies of India), and Ibn Battuta (whose travel writings provided detailed ethnographic accounts) developed methodologies and insights that would later be claimed as "innovations" by European anthropologists and sociologists.

Here, I would like to finish by highlighting that the decolonising movement’s immediate concern is a pivotal, scholarly speaking: that anthropology as a knowledge-making mechanism should be critical about its engagement with power mechanisms, and the mechanisms that generate inequalities and even violence. This critical engagement requires not just incorporating non-Western thinkers into existing theoretical frameworks, but reconsidering how different intellectual traditions can inform our understanding of social theory itself. The case of Ibn Khaldun demonstrates how classical Arab scholarship can contribute to contemporary theoretical debates while avoiding both Eurocentric dismissal and uncritical romanticisation. This suggests a path forward for decolonial approaches that engage deeply with diverse intellectual traditions while maintaining critical theoretical perspectives

## Data Availability

No data are associated with this article.

## Footnotes

^1^ To clarify, the initial translation of Greek texts to non-Greek languages did not happen directly from Greek to Arabic. Most of those resources were first translated to another Middle Eastern-East Mediterranean language, Syriac. In around 700s, those Syriac translations started being translated into Arabic and eventually, the direct translations from original Greek sources started emerging as well.

^2^ Sadly, most of those translations were lost or destroyed during Mongol invasions between 1207 and 1340.

^3^ Although the use of the word Berber is questioned today due to its Roman derogatory reference and Imazighen is instead used to avoid the problem, I will have to follow Ibn Khaldun’s original Arabic text(s) to maintain consistency and surface the critical position I am taking across this text.

^4^ Such inconsistencies however do not exist in languages that engage with this scholarship more closely, such as Persian and Turkish.

^5^ I am not even getting into how west vs Islam are categorically incorrect dualities.
