# Supplementary material for: Genealogy, critique, and decolonisation: Ibn Khaldun and moving beyond filling the gaps
Source: Open Res Eur. 2025 May 16;4:14. Originally published 2024 Jan 10. [Version 2] doi: 10.12688/openreseurope.16148.2 (PMC12177447; doi:10.12688/openreseurope.16148.2)
Supplement: Peer review history

# Reviewer report: Reviewer response for version 2

**DOI:** 10.21956/openreseurope.21945.r54612

**Published:** 2025-07-24

**Authors:** Patrícia Ferraz de Matos

**Affiliations:** 1 University of Lisboa, Lisbon, Lisbon, Portugal

This article addresses a pertinent topic – how anthropology can engage with decolonial thinking – but could be improved in terms of its coverage of the history of the discipline and the reasons that may have led to certain choices and omissions over time.

 In a way, the reader is misled, as the author suggests she will focus on a lesser-known period – between 400 BC and the 1500s – rather than on Ancient Greece, the Enlightenment, and the post-Enlightenment period. Despite providing examples of authors who may be considered part of Classical Arab Scholarship, the reader is not necessarily better informed about the context or the authors that the author says she will analyse.

 In terms of the history of the discipline, it is important to note that anthropology did not simply emerge from contexts related to Western colonisation. In some cases, the discipline emerged independently (studying groups that already existed in different regions of the same country, for example, or groups with distinguishable physical or cultural characteristics), and would have existed even without the colonial context. In other cases, the colonial context also existed, and the discipline emerged stronger, or this context ended up contributing to its reinforcement and/or institutionalisation.

 The article intends to address several different aspects throughout the text, but connections are not always established between these aspects and the subjects indicated at the beginning. Thus, despite the interesting subject matter, the narrative is not always accurate and the author does not make the most of the analysis she proposes.

 The main theme of the article is not clear from the outset. In other words, it would have been helpful if the author had indicated what she was going to study, why, and how (i.e. the methodology and sources used). It would also have been helpful if she had set out a clear set of questions and explained how she intended to answer them.

 Several topics mentioned, such as scientific racism, deserve better contextualisation with more bibliographical references, particularly to support her claims. Although they focus mainly on the Western context, some books edited by George Stocking Jr. (from the History of Anthropology collection, for example) could have helped.

 I would like to draw attention to the use of the word “races” without quotation marks. Although it has been used historically as a differentiator between populations, it is an element that does not exist.

 The concept of “asabiyyah” seems particularly interesting, and the author could have explored it further by drawing parallels with Western concepts, for example. The way the author describe it reminds the 19th century concept of nation described by E. Renan, which also evokes feelings and social cohesion. Would it be worthwhile trying to make comparisons, thus bringing together or clarifying worlds and times that are (perhaps apparently) so distinct?

 I particularly liked the translation aspect, and how a simple author's name can be translated in several different ways. I suggest delving deeper into this aspect of translation and its potential for analysis, as intended by the article.

 Finally, I suggest avoiding the first person in the abstract and using the third person singular instead.

 I also suggest including the name Ibn Khaldun in the keywords, as well as one or two more words that better describe the content of the article. For example, I suggest replacing the word “genealogy” with "genealogy of knowledge" or "dismissed genealogies in anthropological theory", since the article is not about genealogy (a topic also analysed in anthropology), and the current wording may mislead readers.

Is the argument persuasive and supported by appropriate evidence?

Partly

Is the work clearly and cogently presented?

Partly

Is the topic of the essay discussed accurately in the context of the current literature?

Partly

Does the essay contribute to the cultural, historical, social understanding of the field?

Yes

Reviewer Expertise:

Social and Cultural Anthropology; History of Anthropology; History of Science; Anthropology of Colonialism;

I confirm that I have read this submission and believe that I have an appropriate level of expertise to confirm that it is of an acceptable scientific standard, however I have significant reservations, as outlined above.

# Reviewer report: Reviewer response for version 2

**DOI:** 10.21956/openreseurope.21945.r54610

**Published:** 2025-06-24

**Authors:** Karen Sykes

**Affiliations:** 1 The University of Manchester, Manchester, England, UK

1, Yes. The topic is accurately discussed. Sehlikoglu’s argument develops Ghassan Hage’s assertion, “the decolonising movement is about critiquing the canon but, while doing so, creating a more thorough, detailed, stronger and comprehensive genealogy of knowledge.”

 2, Yes. The argument progresses fluidly by pointing out the vagaries of the uses of genealogy as a concept.

 3, Partly. The argument is persuasive and can be supported by even more appropriate evidence. The reference to HAU Facebook polemics is not a strong starting place for a discussion of anthropological genealogies of political society. Sahlins’s main points are developed better in his article, *The original political society*, published in the same year in the journal HAU. 

 Here are some notes for the author’s consideration:

 Sahlins argues that political society in Oceania never divided state from non-state society. States can and did exist without bureaucracies or institutions to support them. He resists evolutionary accounts that propose states emerge from non-state societies. Instead, the ‘idea’ of a non-state society emerges with the state.

 Sahlins’s is concerned with social scientists’ knowledge of ‘non-literate’ intellectual traditions, whereas Khaldun’s theory of social solidarity arises from a literary tradition. This difference is critical for how scholars deploy the concept of genealogy.  

 How does colonialism’s governing and administrative practice make literacy into a powerful tool for the colonisation of knowledge into distinctly different genealogies?

 How do anthropologists bear responsibility for the scholarly record of intellectual traditions not arising from the practices of literacy? 

 4, Partly.  See point 3.

Is the argument persuasive and supported by appropriate evidence?

Partly

Is the work clearly and cogently presented?

Yes

Is the topic of the essay discussed accurately in the context of the current literature?

Yes

Does the essay contribute to the cultural, historical, social understanding of the field?

Partly

Reviewer Expertise:

Anthropology of Oceania; postcolonial education; exchange and lifecycle rituals.

I confirm that I have read this submission and believe that I have an appropriate level of expertise to confirm that it is of an acceptable scientific standard, however I have significant reservations, as outlined above.

# Reviewer report: Reviewer response for version 2

**DOI:** 10.21956/openreseurope.21945.r54615

**Published:** 2025-06-18

**Authors:** Stephen Campbell

**Affiliations:** 1 Nanyang Technological University, Singapore, Singapore

The author appears to have addressed the comments and suggestions from the initial two reviewers. I do not have additional comments or suggestions for revisions at this stage in the review process. I will add that I find this open review process with multiple rounds of reviews by different reviewers after the article has already been published online to be quite strange.

Is the argument persuasive and supported by appropriate evidence?

Yes

Is the work clearly and cogently presented?

Yes

Is the topic of the essay discussed accurately in the context of the current literature?

Yes

Does the essay contribute to the cultural, historical, social understanding of the field?

Yes

Reviewer Expertise:

labour, migrant, borders, Southeast Asia

I confirm that I have read this submission and believe that I have an appropriate level of expertise to confirm that it is of an acceptable scientific standard.

# Reviewer report: Reviewer response for version 1

**DOI:** 10.21956/openreseurope.17432.r38237

**Published:** 2024-03-12

**Authors:** Gennaro Ascione

**Affiliations:** 1 Universita degli Studi di Napoli L'Orientale, Naples, Campania, Italy

The article provides a useful entry point to explore the relevance of one major figure in Social Science - Ibn Khaldun. Such a major figure has remained largely unaddressed since his inclusion in the canon of social sciences disturbs the canonical self-biography of the modern western thinking.

 The article is useful to expand and articulate the premodern Mediterranean space of intellectual dialogue across the taken-for-granted separation between Islam and Christianity, and the cultural formations associated with it.

 The article correctly points to question the automatic association of critical thinking abut Eurocentrism with the semantic of 'decolonization'.

 Given the space of the article, however, some very important questions/results are only skimmed over, and overlooked, giving the sense of a superficial view over a long-term process of historical social change.

 My suggestion is to extend the paragraphs whose titles refer to just one-single page of argumentation, or merge these paragraphs into larger units, in order to give a more consolidated outlook to the entire contribution

Is the argument persuasive and supported by appropriate evidence?

Yes

Is the work clearly and cogently presented?

Yes

Is the topic of the essay discussed accurately in the context of the current literature?

Yes

Does the essay contribute to the cultural, historical, social understanding of the field?

Yes

Reviewer Expertise:

Premodern Mediterranenan intellectual history. Renaissance philosophy, Sociology of Knowledge, Historical Epistemology, Decolonial and Postcolonial Studies, non-western epistemologies, methodologies of human and social sciences, Postcolonial and Decolonial Studies, Global Studies

I confirm that I have read this submission and believe that I have an appropriate level of expertise to confirm that it is of an acceptable scientific standard, however I have significant reservations, as outlined above.

# Reviewer report: Reviewer response for version 1

**DOI:** 10.21956/openreseurope.17432.r38243

**Published:** 2024-03-05

**Authors:** Alaka Wali

**Affiliations:** 1 Field Museum, Chicago, USA

This article is an important contribution to the literature on decolonization in anthropology and how to expand the discourse. The author argues that the "dismissal" of classical Arabic scholarship leaves a considerable gap in knowledge production. He/she uses the work of Ibn Khaldun. who while she/he is acknowledged, has also only recently been considered along with other theoretical thinkers of the Western cannon. At the same time, he cautions against over-romanticizing these classical scholars in a political context that is polarizing and replicates the dichotomies between Western and Non-Western scholarship, when in reality, in Ibn Khaldun's time there was a significant interaction between Greek and Arabic scholars (among others).  He/she also points out that Ibn Khaldun and others were Arabic but not necessarily "Islamic".   

 The case she/he makes is strong but I recommend they strengthen the article by doing some close copy editing (for grammatical and stylistic improvements) , and including more citations (for example on page 9, he states "a number of scholars"... but doesn't provide citations).  Also, the last sections of the article (starting with "Founding a discipline") could be tightened and clarified. The author makes a critical point that there needs to be an nuanced use of Ibn Khaldun's work and not just using it to critique Western theorists (such as Foucault),  but the argument seems to get muddled at times.  The author might want to elaborate on some of the points made in these sections, and perhaps eliminate some digressions such as the spellings of names, etc. and translation issues.

Is the argument persuasive and supported by appropriate evidence?

Partly

Is the work clearly and cogently presented?

Partly

Is the topic of the essay discussed accurately in the context of the current literature?

Yes

Does the essay contribute to the cultural, historical, social understanding of the field?

Yes

Reviewer Expertise:

Cultural Anthropology, decolonization of museum practice

I confirm that I have read this submission and believe that I have an appropriate level of expertise to confirm that it is of an acceptable scientific standard, however I have significant reservations, as outlined above.
